# Supplementary material for: Spiculogenesis and biomineralization in early sponge animals
Source: Nat Commun. 2019 Jul 26;10:3348. doi: 10.1038/s41467-019-11297-4 (PMC6659672; doi:10.1038/s41467-019-11297-4)
Supplement: Supplementary file 1 — Supplementary Information [file 41467_2019_11297_MOESM1_ESM.pdf]

Supplementary Information

Spiculogenesis and biomineralization in early sponge animals

Tang et al.

This file includes:

Supplementary Figures 1 to 4

Supplementary References

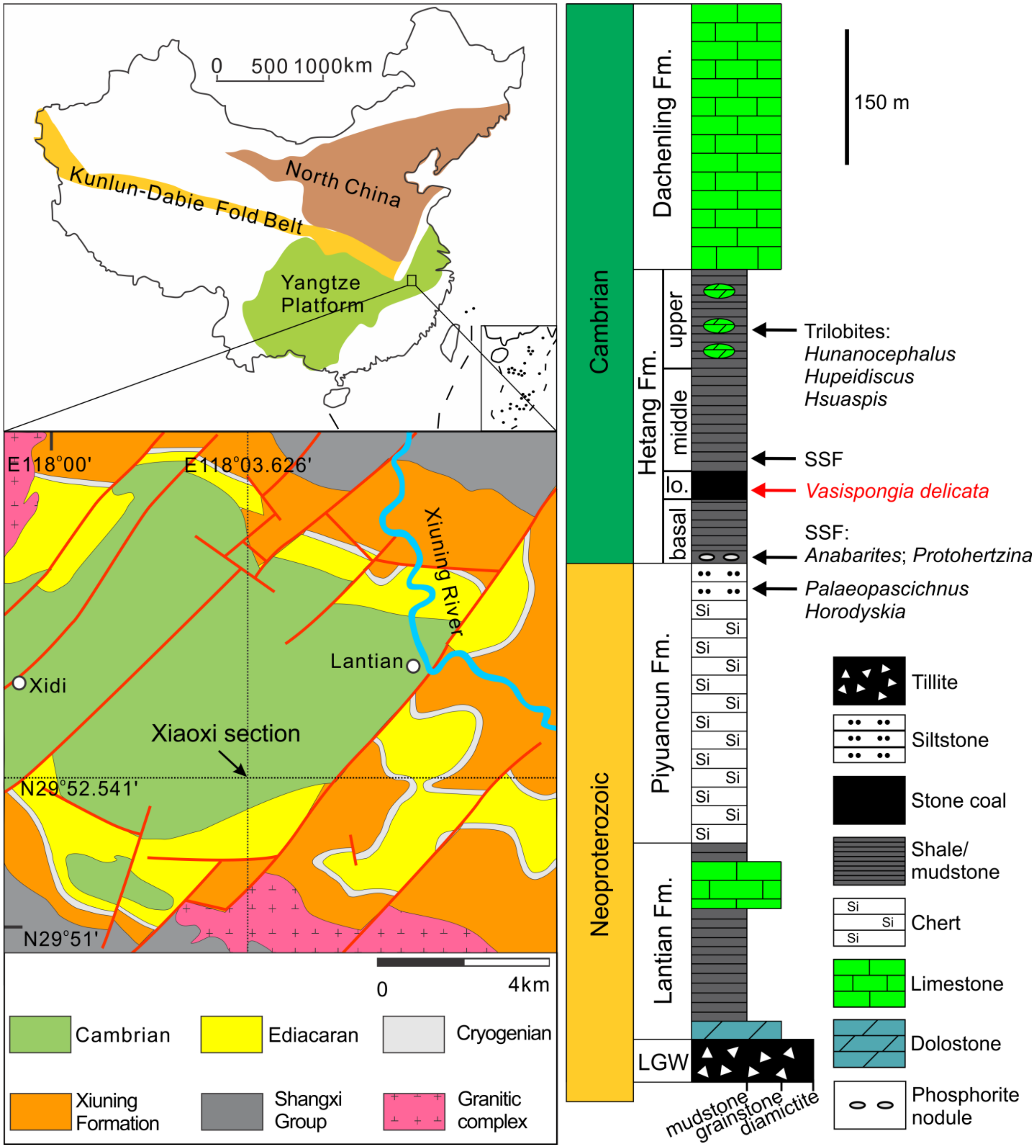

Supplementary Figure 1. Geological map and stratigraphic column of the Neoproterozoic–lower Cambrian in the Lantian area, South China. Modified from refs [1,2]. LGW: Leigongwu Formation, SSF: small shelly fossil, Fm.: Formation.

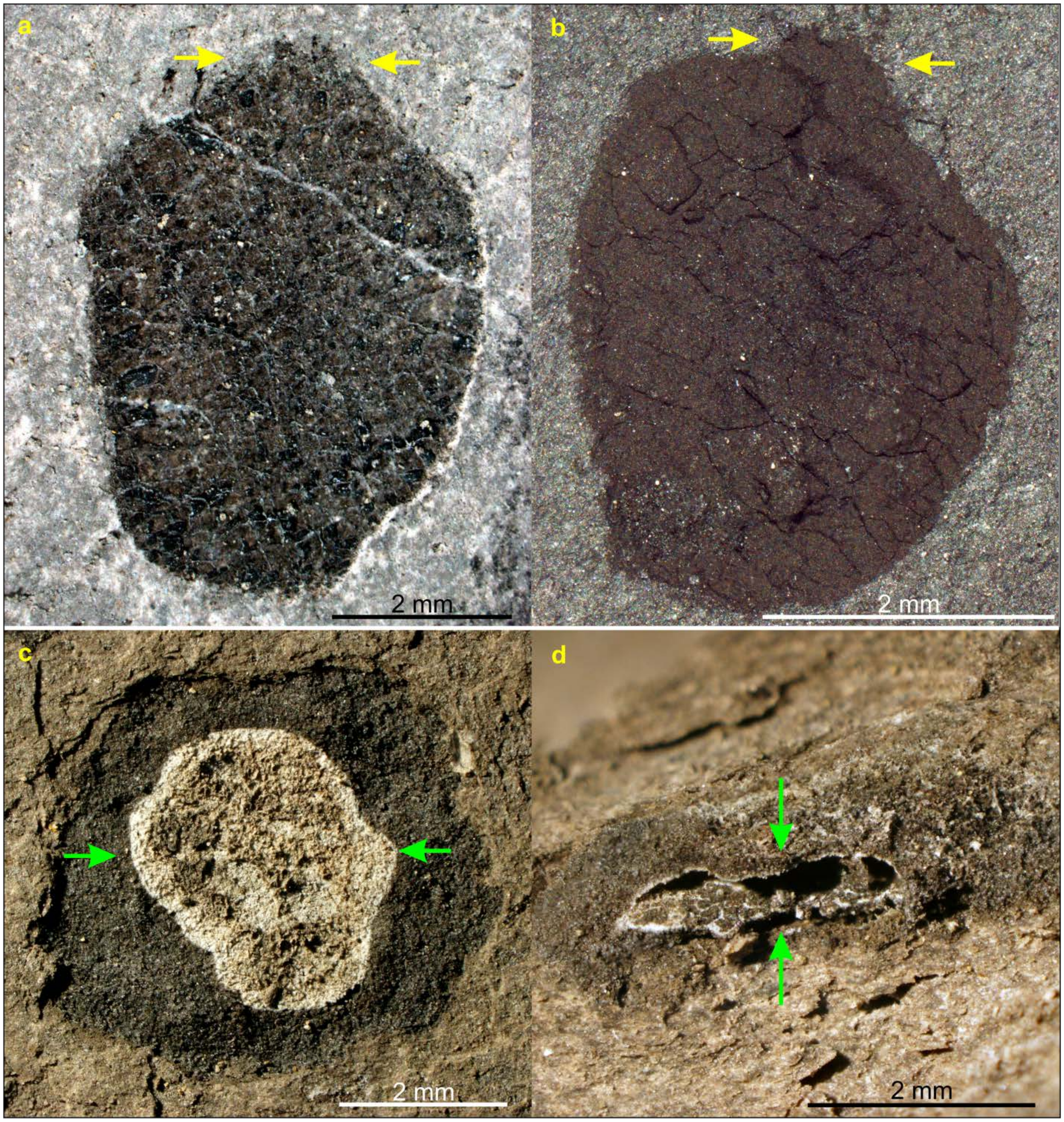

Supplementary Figure 2. ***Vasispongia delicata*** **Tang and Xiao, n. gen. & sp., showing putative oscula and spongocoels. a, b** Sponge fossils with their oscula bracketed by yellow arrows, VPIGM-4725 and VPIGM-4726, respectively. **c**, **d** Sponge fossils with their spongocoels bracketed by green arrows, VPIGM-4727 and VPIGM-4728, respectively. Note that **d** is a cross-sectional view of a less compressed specimen with a three-dimensionally preserved spongocoel.

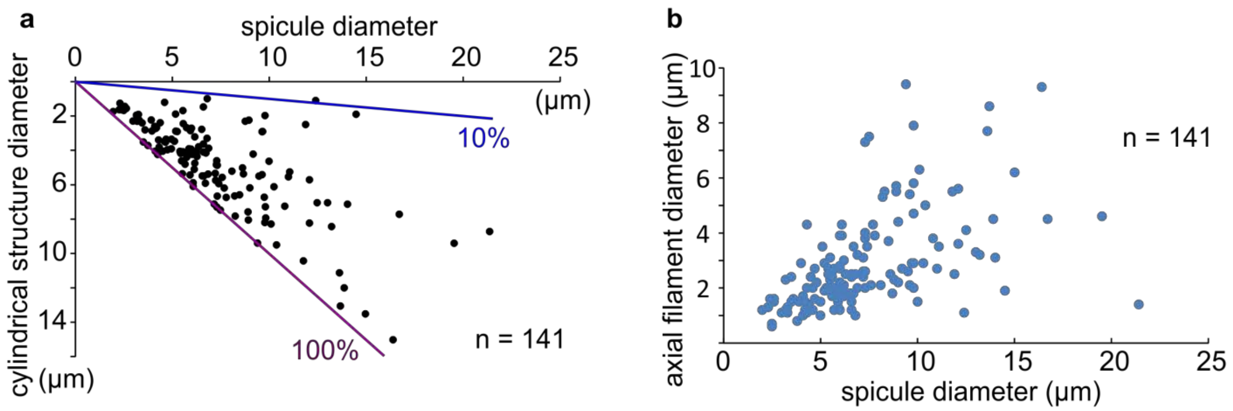

Supplementary Figure 3. Biometric data of *Vasispongia delicata* spicules. a Cross-plot of spicule diameter and cylindrical structure diameter (= inner core diameter + 2 * outer lamella thickness). Organic cylindrical structure accounts for ~10–100% of spicule diameter. **b** Cross-plot of spicule diameter and axial filament diameter. Source data are provided as a Source Data file.

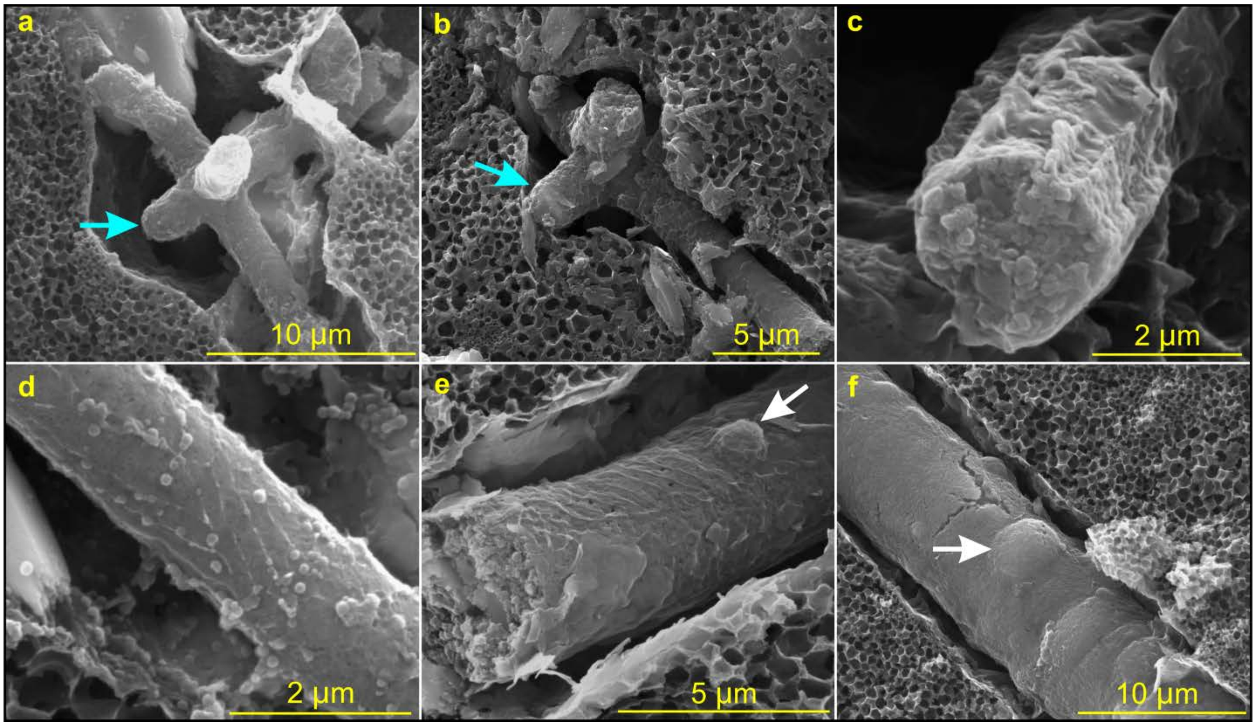

Supplementary Figure 4. Protuberances, ridges, and tubercles on axial filaments (or inner cores) of *Vasispongia delicata* spicules. a, **b** Axial filaments with aborted rays or protuberances (cyan arrows), VPIGM-4719 and VPIGM-4720, respectively. **c, d** Axial filaments with ridges, VPIGM-4721 and VPIGM-4722, respectively. **e** Axial filament with both ridges and a tubercle (white arrow), VPIGM-4723. **f** Axial filament with a tubercle (white arrow), VPIGM-4724. ic: inner core. Honeycomb-like structures in the sedimentary matrix are molds of framboidal pyrite.

Supplementary References

1 Xiao, S., Hu, J., Yuan, X., Parsley, R. L. & Cao, R. Articulated sponges from the Lower Cambrian Hetang Formation in southern Anhui, South China: their age and implications for the early evolution of sponges. *Palaeogeogr. Palaeoclimatol. Palaeoecol.* **220**, 89−117 (2005).

2 Dong, L. *et al.* Micro- and macrofossils from the Piyuancun Formation and their implications for the Ediacaran-Cambrian boundary in southern Anhui. *J. Stratigr.* **36**, 600−610 (2012).
