# Supplementary material for: Spiculogenesis and biomineralization in early sponge animals
Source: Nat Commun. 2019 Jul 26;10:3348. doi: 10.1038/s41467-019-11297-4 (PMC6659672; doi:10.1038/s41467-019-11297-4)
Supplement: Supplementary file 3 — Reporting Summary [file 41467_2019_11297_MOESM3_ESM.pdf]

nature research

Corresponding author(s): Shuhai Xiao
Last updated by author(s): May 21, 2019

Reporting Summary

Nature Research wishes to improve the reproducibility of the work that we publish. This form provides structure for consistency and transparency in reporting. For further information on Nature Research policies, see Authors & Referees and the Editorial Policy Checklist .

Statistics

For all statistical analyses, confirm that the following items are present in the figure legend, table legend, main text, or Methods section.

n/a	Confirmed	
[ ]	[x]	The exact sample size (*n*) for each experimental group/condition, given as a discrete number and unit of measurement
[ ]	[x]	A statement on whether measurements were taken from distinct samples or whether the same sample was measured repeatedly
[ ]	[x]	The statistical test(s) used AND whether they are one- or two-sided<br>*Only common tests should be described solely by name; describe more complex techniques in the Methods section.*
[x]	[ ]	A description of all covariates tested
[x]	[ ]	A description of any assumptions or corrections, such as tests of normality and adjustment for multiple comparisons
[ ]	[x]	A full description of the statistical parameters including central tendency (e.g. means) or other basic estimates (e.g. regression coefficient) AND variation (e.g. standard deviation) or associated estimates of uncertainty (e.g. confidence intervals)
[ ]	[x]	For null hypothesis testing, the test statistic (e.g. *F*, *t*, *r*) with confidence intervals, effect sizes, degrees of freedom and *P* value noted<br>*Give P values as exact values whenever suitable.*
[x]	[ ]	For Bayesian analysis, information on the choice of priors and Markov chain Monte Carlo settings
[x]	[ ]	For hierarchical and complex designs, identification of the appropriate level for tests and full reporting of outcomes
[x]	[ ]	Estimates of effect sizes (e.g. Cohen's *d*, Pearson's *r*), indicating how they were calculated

Our web collection on statistics for biologists contains articles on many of the points above.

Software and code

Policy information about availability of computer code

Data collection	Image J
Data analysis	Microsoft Excel 2010; JMP

For manuscripts utilizing custom algorithms or software that are central to the research but not yet described in published literature, software must be made available to editors/reviewers. We strongly encourage code deposition in a community repository (e.g. GitHub). See the Nature Research guidelines for submitting code & software for further information.

Data

Policy information about availability of data

All manuscripts must include a data availability statement. This statement should provide the following information, where applicable:

- Accession codes, unique identifiers, or web links for publicly available datasets
- A list of figures that have associated raw data
- A description of any restrictions on data availability

All measurement data are included in the supplementary information of the manuscript. Fossil repository information is provided in the manuscript.

Field-specific reporting

Please select the one below that is the best fit for your research. If you are not sure, read the appropriate sections before making your selection.

[ ] Life sciences [ ] Behavioural & social sciences [x] Ecological, evolutionary & environmental sciences

For a reference copy of the document with all sections, see nature.com/documents/nr-reporting-summary-flat.pdf

Ecological, evolutionary & environmental sciences study design

All studies must disclose on these points even when the disclosure is negative.

Study description	Palaeontological study of sponge fossils collected from the early Cambrian Hetang Formation in Lantian area, southern Anhui Province of China.
Research sample	Fossil specimens were collected from the stone coal member of the Hetang Formation at Xiaoxi section (29°52.541'N, 118°03.626'E) in the Lantian area of Anhui Province, China. All specimens described in this paper are reposited at Virginia Polytechnic Institute Geosciences Museum. Accession numbers of illustrated specimens are provided in the manuscript.
Sampling strategy	Sponge specimens were collected from outcrops with stratigraphic horizon noted. They were then shipped to the Virginia Tech where they were photographed, measured, and analyzed.
Data collection	Measurements were made on photographs using Image J.
Timing and spatial scale	Fossils were collected in 2016 from stratigraphic horizons about 70-80 m above the base of the Hetang Formation at the Xiaoxi section (29°52.541'N, 118°03.626'E) in the Lantian area of Anhui Province, China.
Data exclusions	No data were excluded from analysis.
Reproducibility	To ensure reproducibility, multiple specimens with the same structure are measured. The raw data are presented in Supplementary Information.
Randomization	None.
Blinding	None.

Did the study involve field work? [x] Yes [ ] No

Field work, collection and transport

Field conditions	The field site is located in the subtropical zone. Climate is humid in the field season (summer time). Outcrops are well exposed. Excavation was required to remove slab of fossil specimens.
Location	Xiaoxi section (29°52.541'N, 118°03.626'E) in the Lantian area of Anhui Province, China.
Access and import/export	Collection of fossil specimens was carried out in a responsible manner and in compliance with the local, national and international laws. Specimens are publicly accessible at the Virginia Tech, with accession numbers provided in the manuscript.
Disturbance	None.

Reporting for specific materials, systems and methods

We require information from authors about some types of materials, experimental systems and methods used in many studies. Here, indicate whether each material, system or method listed is relevant to your study. If you are not sure if a list item applies to your research, read the appropriate section before selecting a response.

Materials & experimental systems

n/a	Involved in the study	
[x]	[ ]	Antibodies
[x]	[ ]	Eukaryotic cell lines
[ ]	[x]	Palaeontology
[x]	[ ]	Animals and other organisms
[x]	[ ]	Human research participants
[x]	[ ]	Clinical data

Methods

n/a	Involved in the study	
[x]	[ ]	ChIP-seq
[x]	[ ]	Flow cytometry
[x]	[ ]	MRI-based neuroimaging

Palaeontology

Specimen provenance	Stone coal member of the Hetang Formation, Xiaoxi section (29°52.541'N, 118°03.626'E) in the Lantian area of Anhui Province, China.
Specimen deposition	All specimens described in this paper are reposited at Virginia Polytechnic Institute Geosciences Museum. Accession numbers of illustrated specimens are provided in the manuscript.
Dating methods	Published biostratigraphic correlation with early Cambrian strata in South China (Xiao et al., 2005) are used to constrain the age of the fossils.

☒ Tick this box to confirm that the raw and calibrated dates are available in the paper or in Supplementary Information.
