# Supplementary material for: Spiculogenesis and biomineralization in early sponge animals
Source: Nat Commun. 2019 Jul 26;10:3348. doi: 10.1038/s41467-019-11297-4 (PMC6659672; doi:10.1038/s41467-019-11297-4)
Supplement: Supplementary file 4 — Description of Additional Supplementary Files [file 41467_2019_11297_MOESM4_ESM.pdf]

Description of Additional Supplementary Files

File Name: Supplementary Data 1

Description: **Measurements of Vasispongia delicata and its constituent spicules.** "0" in the column of "organic layer thickness (t_{ol})" denotes that an organic layer is not preserved or developed. s.d. = standard deviation; d_s = spicule diameter; d_{af} = axial filament diameter; t_{ol} = organic layer thickness; r_{op} = organic proportion. Axial filament as a proportion of spicule diameter is represented by a ratio = (d_{af} /$_{ds}$) * 100%. The relative organic proportion of the spicules is represented by a ratio rop = ((d_{af} + 2 * t_{ol}) /d_s) * 100% (plotted as purple symbols in Fig. 6).

File Name: Supplementary Data 2

Description: **Measurements of fossil and extant spicules of representative poriferan groups/classes.** d_s = spicule diameter; d_{af} = axial filament diameter; t_{ol} = organic layer thickness; tos = outer sheath thickness; dac = axial canal diameter; rop = organic proportion. “×” denotes that the structure is not developed or preserved; “√” denotes that the structure is present but unable to measure due to poor preservation or illustrations. The relative organic proportion of sponge spicules is quantified as a ratio: when the axial filaments are preserved, this ratio was calculated as rop = (d_{af} + (t_{ol} + t_{os}) * 2)/d_s * 100% (plotted as orange symbols in Fig. 6); when only the axial canal is preserved, this ratio was calculated as rop = (d_{ac} + (t_{ol} + t_{os}) * 2)/d_s * 100% (plotted as gray symbols in Fig. 6). Because axial canals can be enlarged by mineral dissolution1, the latter method and the gray symbols in Fig. 6 represent maximum estimates of organic proportion in sponge spicules. The absolute age of fossil occurrences is an average between the maximum and minimum age constraints unless otherwise available in the referred publications. For example, the Hetang Formation is regarded as Cambrian Stage 2 (~529−521 Ma) and the Hetang sponge fossils are estimated to be ~525 Ma.
